# Estrogen Effects on Wound Healing

**DOI:** 10.3390/ijms18112325

**Published:** 2017-11-03

**Authors:** Huann-Cheng Horng, Wen-Hsun Chang, Chang-Ching Yeh, Ben-Shian Huang, Chia-Pei Chang, Yi-Jen Chen, Kuan-Hao Tsui, Peng-Hui Wang

**Affiliations:** 1 Department of Obstetrics and Gynecology, Taipei Veterans General Hospital, Taipei 112, Taiwan; hchorng@vghtpe.gov.tw (H.-C.H.); whchang@vghtpe.gov.tw (W.-H.C.); ccyeh39@gmail.com (C.-C.Y.); benshianhuang@gmail.com (B.-S.H.); jpchang2@vghtpe.gov.tw (C.-P.C.); chenyj@vghtpe.gov.tw (Y.-J.C.); 2 Department of Obstetrics and Gynecology, National Yang-Ming University, Taipei 112, Taiwan; khtsui60@gmail.com; 3 Institute of BioMedical Informatics, National Yang-Ming University, Taipei 112, Taiwan; 4 Department of Nursing, Taipei Veterans General Hospital, Taipei 112, Taiwan; 5 Department of Nursing, National Yang-Ming University, Taipei 112, Taiwan; 6 Institute of Clinical Medicine, National Yang-Ming University, Taipei 112, Taiwan; 7 Department of Obstetrics and Gynecology, Kaohsiung Veterans General Hospital, Kaohsiung 813, Taiwan; 8 Department of Pharmacy and Graduate Institute of Pharmaceutical Technology, Tajen University, Pingtung County 900, Taiwan; 9 Department of Medical Research, China Medical University Hospital, Taichung 404, Taiwan

**Keywords:** estrogen, estrogen receptor, wound healing

## Abstract

Wound healing is a physiological process, involving three successive and overlapping phases—hemostasis/inflammation, proliferation, and remodeling—to maintain the integrity of skin after trauma, either by accident or by procedure. Any disruption or unbalanced distribution of these processes might result in abnormal wound healing. Many molecular and clinical data support the effects of estrogen on normal skin homeostasis and wound healing. Estrogen deficiency, for example in postmenopausal women, is detrimental to wound healing processes, notably inflammation and re-granulation, while exogenous estrogen treatment may reverse these effects. Understanding the role of estrogen on skin might provide further opportunities to develop estrogen-related therapy for assistance in wound healing.

## 1. Introduction

The epidermis and dermis of normal skin maintain a steady-state equilibrium to ensure a protective barrier against the external environment [1,2,3]. When the protective barrier is broken by trauma, injury, radiation, chemical injury, and/or burns, the wound healing process is immediately set in motion and complex biochemical events take place to repair the damage [4,5,6,7,8]. The normal wound healing process involves three successive and overlapping stages, including the hemostasis/inflammation phase, the proliferation phase, and the remodeling phase [9,10,11,12,13,14,15].

After an injury to skin, hemostasis activates immediately. The exposed sub-endothelium, collagen, and tissue factor will activate platelet aggregation, which leads to degranulation and releasing chemotactic and growth factors (GF), such as proteases, platelet-derived GF, and vasoactive agents (histamine and serotonin) [16]. Clot formation is obtained, thus achieving successful hemostasis. Chemokines released by platelet activation arouse chemotaxis of neutrophil granulocytes, macrophages, and T lymphocytes into the wound to cleanse debris and bacteria to provide a desired environment for wound healing [16]. Neutrophils are the first cells to appear at the injury site, and create an environment to prevent infection [10,11]. Then, macrophages accumulate within the injury sites and facilitate the phagocytosis of bacteria and damaged tissue. The hemostasis/inflammatory phase proceeds for 48 to 72 h and then the proliferative phase follows.

The proliferative phase, represented by profuse fibroblasts, keratinocytes, endothelial cells, and an accumulation of extracellular matrix (ECM), attenuates gradually and lasts for three to six weeks [10,11]. During the proliferation phase, granulation tissue is formed to replace the clot formation originally created after injury. The major components of granulation tissue are macrophages, fibroblasts, proteoglycans, hyaluronic acid, collagen, and elastin. Additionally, the recruited fibroblasts differentiate into myofibroblasts to achieve wound contraction by cytokines and/or molecules and GFs, including platelet-derived GF, fibroblast GF, nerve GF, transforming GF-β (TGF-β, including TGF-β1, TGF-β2, and TGF-β3), connective tissue GF (CTGF), cysteine-rich 61 (Cyr61), interleukins (IL, including IL-6, IL-8, and IL-10), homeobox 13, the Wnt signaling pathway, osteopontin, and early growth response protein 1 [6,7,8,17,18,19,20,21,22,23,24]. The upregulation of vascular endothelial GFs all provide a structural framework for endothelial cells to proliferate and lay down new vessels by the process of angiogenesis during this phase [10,22,23]. Re-epithelialization is essential for the re-establishment of tissue integrity. After the completion of re-epithelialization, represented by keratinocytes from wound edge and neighboring adnexal structures migrating across the wound surface, apoptosis of myofibroblasts then occurs, and the skin defect is closed.

The subsequent remodeling phase often takes six to nine months or up to a year to complete [7,8,9,10,13]. During the remodeling phase, a precise balance between the apoptosis of existing cells and the production of new cells should be fulfilled. Profuse ECM is degraded gradually and the immature type III collagen is amended into mature type I collagen, resulting in stronger scar tissue. The following are critical for normal wound healing: the orientation of type I collagen, the regression of the immature vessels, and the activation of myofibroblasts [10,25,26,27]. The final actin-rich cells and reorganization of mature blood contribute to normal wound healing.

Many factors play a role in wound healing [3,4,7,8,9,10,11,12,13,14,15,16,17,18,19,20,21,22,23,24,28,29,30], including endocrine factors. The aims of the current review are limited mainly to the discussion of the effects of estrogen on wound healing.

## 2. Estrogen and Estrogen Signaling

The three major forms of estrogens in humans are estradiol, estrone, and estriol, with estradiol being the predominant form [31,32,33,34,35]. The ovary is the main source of estrogen in the premenopausal period, and granulosa cells are the key cells to produce estrogen [36,37,38,39,40]. Estrogen is also synthesized in skin tissue [41], presenting a concept termed “intracrinology” [42]. Crucially, estrogen regulation depends on estrogen-metabolism enzymes, which include aromatase, catalyzing the conversion of androgens (including dehydroepiandrosterone (DHEA) and testosterone) to estrogens, as well as 17β-hydroxysteroid dehydrogenase 1 and 17β-hydroxysteroid dehydrogenase 2 converting estrone to estradiol [42].

Many natural or chemical synthetic compounds imitating estrogen have been found, and we called these compounds estrogen-like compounds, such as selective estrogen receptor modulators (SERMs), phytoestrogens, and others [31,43,44,45]. The action of these estrogen-like compounds on cells or tissues is far more complicated, although the main pathway might follow the classical ligand-mediated nuclear receptor signaling, such as estrogen receptors α and β (ERα and ERβ) located within the nuclei of target cells [46,47]. The absence of a ligand (estrogen) shows that ERs are associated with an inhibitory heat shock protein [41].

Estrogen receptors, class I members of the superfamily of nuclear receptors characterized as ligand-inducible transcription factors, have considerable sequence identity in the DNA-binding domains, which permits both receptor types (homo- or hetero-dimers) to interact with the ER elements of various genes. Sequence differences between the two receptors occur primarily in the N- and C-terminal regions. Estrogen receptors can also act as coregulators through binding to other transcription factors already attached to the gene regulatory region and by ligand-independent mechanisms [31,43]. Furthermore, each ER has several known isoforms; at least two isoforms of the gene product relating to ERα and six isoforms of the gene product relating to ERβ are known [31]. ERs are identified in skin, but the expression of ERs on skin depends on the location in the body and cell type [42].

In addition of classical estrogen and ER action, membrane-bound ERs show a distinct role [48]. G-protein-coupled ER1 (GPER1) or G-protein-coupled receptor 30 (GRP30) is a seven-transmembrane receptor protein that binds specifically to estrogen and plays an important role in rapid non-genomic cell signaling [48,49]. Taken together, estrogen signaling can be mediated either through a rapid non-genomic effect or a classical genomic effect. The intricate biochemistry of the mode of action of ERs can be separated into three major aspects, including: (1) the interaction of ERs with other membrane-bound receptors as a form of non-genomic action to regulate the activation or suppression of different transcription factors; (2) the mechanism of ER action alone or in combination of other transcription factors as a form of genomic action to modulate DNA-binding ability to influence gene expression; (3) specific amino acid sequences and structural configurations of ERs [48].

## 3. Estrogen Signaling and Wound Healing

To explore the relationship between estrogen and wound healing, the term “wound healing and oestrogen” (from 1947 to 30 July 2017) was used to search PubMed for relevant English-language articles (Available online: https://www.ncbi.nlm.nih.gov/pubmed/?term=wound+healing%2C+estrogen) [50], which identified 532 articles. The following is a brief summary.

The influence of estrogen on wound healing was investigated in the animal model in 1947 [50] and in humans in 1953 [51]. Afterwards, much evidence supports that the estrogen is important on wound healing [31,41,42,52,53,54,55,56,57,58,59,60,61]. It has been shown that primary estrogen deficiency contributes to cutaneous aging and delayed wound repair or impaired wound healing [41,42]. A decrease in the collagen content (both type I and especially type III) of thigh skin in postmenopausal women without hormone replacement therapy has been found at a rate of 2% (skin wrinkling) per postmenopausal year [56]. Compared with premenopausal women, postmenopausal women demonstrate a decrease in collagen types I and III and a reduction in the type III/type I ratio within the dermis, and this change is much more related with the period of estrogen deficiency rather than chronological age [57].

Skin elasticity correlates negatively with years since menopause [56]. Estrogen therapy can rescue the above with an increasing elasticity of 5% over a year [56]. The daily administration of topical estradiol to the skin of postmenopausal women has shown that the amount of hydroxyproline is significantly increased and a morphologic examination by electron microscopy showed a significant improvement in elastic and collagen fibers [58]. Systemic oral estrogen replacement therapy also showed the similar positive effect on skin contour due to the significantly increasing amount of collagen fibers [59].

Microarray-based profiling of genes differentially expressed in rapid and slow wound healing identified 83% of downregulated gene sets and 80% of upregulated probe sets that were estrogen-regulated [60]. Among these, many factors have been investigated, including estrogen-regulated protease inhibitors, arginase 1 (Arg1), and macrophage migration inhibitory factor (MIF) [60,61,62]. These protease inhibitors, including SERPINB4 and SERPINB7, which act to protect against the inappropriate activation of cathepsins, are important for wound healing [60]. Deprivation of these protease inhibitors will result in tissue breakdown [60]. Arg1-expressing cells were significantly decreased in the wound granulation tissue of mice after oophorectomy. Arg1 metabolizes L-arginine to generate proline, a substrate for collagen synthesis. Hence, Arg1 is central to modulating the balance between inflammation and matrix deposition during wound healing [60]. MIF is further discussed in the next section (estrogen signaling on hemostasis/inflammation process) [62]. All of this hints that estrogen has a more profound influence on aging than previously thought.

Additionally, estrogen can protect against the development of a chronic wound [52,60]. Furthermore, estrogen replacement and topical application of estrogen or its precursor accelerate wound healing [53,54,55,61], since systemic DHEA levels are strongly associated with protection against chronic venous ulceration in humans. Women who received hormone replacement therapy were less likely to develop a venous leg ulcer (age-adjusted relative risk (RR) 0.65, 95% confidence interval (CI) 0.61–0.69) or a pressure ulcer (RR 0.68, 95% CI 0.62–0.75) than those who did not use hormone replacement therapy, according to a case-cohort study in the UK General Practice Research Database [63]. Moreover, anti-estrogen therapy, such as tamoxifen or aromatase inhibitors, which plays a major role in hormone receptor-positive breast cancer management [64,65,66], is also correlated with poor wound healing [66]. A study found that women who received anti-estrogen therapy experienced more wound healing complications (61% vs. 28%, *p* < 0.001), fat necrosis (26% vs. 8.3%, *p* < 0.001), infections (15% vs. 2.8%, *p* < 0.001), delayed wound healing (49% vs. 13%, *p* < 0.001), and grade III/IV capsular contracture (55% vs. 9.1%, *p* = 0.001) than those who did not [67], suggesting the important role of estrogen in the wound healing process.

The new insights on the molecular mechanisms mediating the estrogen protective function on wound healing are discussed and topics addressing the involvement of estrogen signaling on hemostasis/inflammation process, proliferation process, and final remodeling process are shown in the following sections.

## 4. Estrogen Signaling on Hemostasis/Inflammation Process

The recognition of the importance of estrogen in skin physiology would suggest it that may have an important role in wound healing, and a number of studies are available to provide evidence that estrogen might have an important role in all phases of wound healing by modifying the inflammatory reaction, accelerating re-epithelialization, stimulating granulation formation, regulating proteolysis, and balancing collagen synthesis and degradation [68,69,70].

The first step of wound healing is immediate hemostasis and a prompt initiation of the inflammatory process. These conditions are mediated by local activation of the coagulation system, hematopoietic system, inflammatory cells, and immune system. Coagulation systems, including coagulation factors, are significantly influenced by estrogen signaling transduction [71,72,73]. However, current knowledge on the effects of estrogen on hemostasis shows the different effect on the coagulation system when the administration route of estrogen is different [73]. A hemostasis imbalance was found among oral estrogen users with a decrease in coagulation inhibitors and an increase in markers of activation coagulation, leading to a global enhanced thrombin formation; by contrast, transdermal estrogen use (avoiding the first-pass effect of liver metabolism) was associated with less change in hemostasis variables and did not activate coagulation and fibrinolysis [73]. Therefore, estrogen deficiency might slow down the activation of coagulation and subsequently impair the immediate hemostasis, which initiates the wound healing process, suggesting the important role of estrogen in hemostasis during wound healing.

During the following inflammation process, the aggregation of megakaryocytes, leukocytes, monocytes, macrophages, lymphocytes, and mast cells is needed. These immune cell populations, including monocytes, neutrophils, macrophages, lymphocytes, and mast cells, as well as hematopoietic progenitors in bone marrow express ERs, suggesting that estrogen directly affects the functions of these cells, including cytokines, in growth factor production [74,75,76,77,78,79]. Even CD34^+^ hematopoietic stem cells in human adult bone marrow, but not in hematopoietic stem cells from cord blood, also express ERs, suggesting that ER expression is highly regulated in hematopoietic precursor development [77]. Estrogen receptor activity augments and dampens innate immune signaling pathways in dendritic cells and macrophages [74].

Monocyte-derived dendritic cells express high levels of ERα and low levels of ERβ [77]. B lymphocytes and plasmacytoid dendritic cells display the highest levels of ERβ in comparison with any other cell type, although B lymphocytes also express the highest levels of ERα compared to other leukocytes [77]. Furthermore, macrophages predominantly express the N-terminal truncated ERα46 protein, and this response is mainly dependent on estrogen induction [78,79]. Since ERα acts directly on hematopoietic stem cells, lymphoid progenitors and myeloid progenitors promote development pathways [78].

Excessive neutrophil recruitment and protease production is often associated with impaired wound healing [68]. Estrogen therapy can reduce the number of wound neutrophils, and reduce the neutrophil adhesion molecule l-selectin, leading to diminished neutrophil localization at sites of inflammation [41]. Administration of estrogen can increase wound fibronectin levels, as well as reduce elastase activity and lessen the degradation of fibronectin in wound tissue [79,80,81].

Estrogen signaling pro-inflammatory cytokine production is varied by the cell type and local estrogen concentration (environmental factors). One report showed that treatment with estrogen can induce ERα expression in macrophages, but the ERβ expression is not changed, and the increasing ERα expression is time-dependent during monocyte-to-macrophage differentiation [82]. The C-X-C motif chemokine ligand 8 (CXCL-8)—one of the chemokines known to cause allergic reactions and profound inflammation [83]—production of macrophages is suppressed in estrogen-mediated ER-dependent pathways.

Additionally, MIF—one of the pro-inflammatory cytokines, which was established by micro-array techniques to identify more than 600 differently regulating repair/inflammation-associated gene targets—is a key player in skin biology and wound healing [60,61,62]. MIF has a direct effect on the expression of genes involved in all aspects of the wound healing process, in addition to genes associated with cellular proliferation, differentiation, and apoptosis in a wide range of cell types [62]. Impaired healing in the absence of estrogen via elevated MIF is associated with dysregulated differentiation, cell contractile machinery, and altered signaling and transcription, coupled with a proteolytic and a pro-inflammatory state [62]. Estrogen treatment can downregulate MIF expression, resulting in an improvement in wound healing [62]. Furthermore, in vitro studies suggested a direct effect on specific pro-inflammatory cytokine production by macrophages via mitogen-activated kinase (MAP), phosphatidylinositol 3 (PI3) kinase pathways, and a nuclear factor κB-dependent mechanism [60,61,62]. This information suggests the importance of ERα expression and regulation in the ability of estrogen to modulate innate immune response.

There are several possible mechanisms to address the relationship between the function of these innate immune cells and estrogen, including estrogen signaling epigenetic changes of immune cells, especially for precursor cells, which might influence downstream developmental pathways or functional responses in mature immune cells. For example, estrogen signaling gene activation promotes a developmental pathway, or altered activity of estrogen signaling transduction in immune cells within a pathway when they are activated [74].

To summarize during the inflammatory phase of wound healing, neutrophils are the first responders of the inflammatory response, and function mainly by clearing debris and pathogens; subsequently, activated macrophages join and continue to clear debris and pathogens, as well as clear apoptotic neutrophils that remain at the wound site. All of them are influenced by estrogen, supporting the important roles of estrogen-mediated signaling transduction in the first step of the wound healing process. Estrogen results in dampening purulent inflammation, decreasing neutrophil numbers as shown above, promoting alternative macrophage polarization (promoting a shift from M1 to M2 subtypes), reducing the expression of pro-inflammatory cytokines, such as tissue necrotic factor (TNF) α, and decreasing elastase production, contributing to the beneficial roles of aiding wound closure and collagen deposition. The role of macrophage phenotypes on wound healing has been extensively reviewed recently [83,84].

In brief, monocytes can be classically or alternatively activated to form M1 and M2 macrophages, respectively [83]. Conventionally, M1 macrophages, which are activated by pro-inflammatory mediators interferon-γ (IFN-γ), TNF, and damage-associated pattern molecules, have a known scavenger function [84]. These classically activated M1 macrophages prolifically produce pro-inflammatory cytokines, such as TNF and IL-6 and other mediators, enabling them to phagocytose neutrophils that have undergone apoptosis and remove any pathogens or debris in the wound. All of this facilitates the initial stages of wound healing. M2 macrophages are typically anti-inflammatory and regulate re-vascularization and wound closure [83]. M2 macrophages can been further divided into four discrete types: M2a, M2b, M2c, and M2d, based on their function and key markers [83]. Collectively, alterations in macrophage number and phenotype will disrupt the wound healing.

Although estrogen is important for wound healing, the effects of estrogen on wounds should be a balancing act, since both low and high levels of estrogen will slow inflammation, and over-inhibition of inflammation is not good for wound healing.

## 5. Estrogen Signaling on the Proliferation Process

The proliferative process of wound healing involves profuse fibroblasts, keratinocytes, and endothelial cells, as well as an accumulation of ECM [10,11]. Therefore, the relationship between estrogen and targeted cells, including fibroblasts, keratinocytes, and endothelial cells, is discussed. All these cells contain ERs.

Cells with low-level differentiation potential, for example mesenchymal cells, have the ability to stimulate tissue renewal. The fibroblast is the key mesenchymal cell type in connective tissue and deposits the collagen and elastic fibers of the ECM, critical for wound healing. Aging and estrogen deficiency result in defects in fibroblast differentiation and functionality associated with impaired hyaluronan synthase 2 and epidermal growth factor receptor function, as a result of upregulated microRNA-7 expression, which mediates the over-activation of JAK/STAT1 [53].

Estrogen signaling interacts with the microRNA-7 promoter, suppresses microRNA-7 expression, and further attenuates STAT1 expression and activity. Estrogen is important for the proliferation, migration, and differentiation of fibroblasts [53], which is primarily mediated by ERα [67]. Estrogen induces a rapid re-organization of the cytoskeleton in dermal fibroblast via the non-classical receptor GPR30, MAPK, PI3 K/Akt, and ERK1/2 activation [48,49,85,86,87]. Finally, the function of fibroblasts may be markedly varied even within a single tissue [87], which results from different autocrine (intracrine) signals. Aromatase activity and Wnt-regulated signals form the overlying epidermis can act both locally, via ECM deposition, and via secreted factors. All impact the behavior of fibroblasts in different dermal locations.

Additionally, human dermal fibroblasts can be “transiently” activated to go forward to myofibroblast differentiation (αSMA expression) [88,89]. Permanent or sequential presence of TGF-β1 and IL-10 might modify the proliferation and migration of fibroblasts and their activated form-myofibroblasts [90]. Study has shown that the removal of TGF-β1 after initial stimulation resulted in an increase of apoptosis of myofibroblasts [90]. TGF-β1 stimulation followed by IL-10 treatment did not result in increased cell apoptosis, but instead led to a significant increase of cell motility and a reduction of myofibroblasts [90]. All of this hints at the precise and dynamic function of cytokines—such as TGF-β1 and IL-10—as an important cue for the completion of wound healing [90].

Keratinocytes proliferate and migrate over the wound to create a barrier between the outer and inner environments, mediated through re-epithelialization. Similar to the impact of estrogen on fibroblasts, estrogen also imparts potent mitogenic effect on keratocytes, promoting in vitro and in vivo migration [41], which might be affected by the estrogen-mediated ERβ interaction with keratocytes. One study showed that the pharmacological activation of ERβ, but not that of ERα, led to a significant alteration in the pattern of differentiation and the proliferation activity of keratinocytes, suggesting that the stimulation of epidermal regeneration may ensue after treating a wound with a targeting to ERβ [91]. However, although the activation of the TGF-β pathway is critical for fibroblast transformation to myofibroblasts [90], the TGF-β pathway is downstream of ERα and ERβ; thus, the ER-mediated function might be opposite [92].

The relation of estrogen to endothelial cells has been investigated before [93,94]. Mature endothelial cells are derived from endothelial progenitor cells from bone marrow, and these endothelial progenitor cells are defined as cells that have a positive receptor to hematopoietic stem cell marker-CD34, endothelial cell marker-vascular endothelial growth factor receptor 2, and an immature hematopoietic stem cell marker CD133 [95]. Estrogen signaling induces the mobilization of circulating endothelial progenitor cells from bone marrow, and these cells help to build and restore injured and/or damaged endothelium. Estrogen also induces proliferation and migration, and inhibits the apoptosis of endothelial progeny cells. The main mechanism of estrogen-mediated re-reendothelialization is a nitric oxide-dependent pathway [93,95]. Endothelial cells constitute an essential cornerstone in the building and maintenance of endothelial blood vessels (lining the lumen of every blood vessel). Endothelial cells are involved in filtration, hemostasis, barrier function, inflammation, and angiogenesis, serving as gatekeepers, preventing degeneration, and assisting in wound healing [95,96].

## 6. Estrogen Signaling on the Remodeling Process

The last step of the wound healing is the remodeling phase, which relies on a controlled balance between the synthesis and degradation of the ECM, and estrogen is thought to involve both [67]. Collagens, composing three α chains of repeating Gly-Xaa-Yaa triplets, which not only induce each α chain to adopt a left-handed PolyPro II helix, but also represent a base by which to classify collagen types, are the most abundant proteins in the human body and the main components of the ECM [96]. Thus far, at least 28 types of collagens have been reported [97]. Collagens can be classified as homotrimeric and heterotrimetric types. Homotrimeric collagens (e.g., type II and type III) have three α chains of identical sequence, and heterotrimetric collagens have two α chains of identical sequence (α1) and one α chain of differing sequence (α2) (i.e., type I), or three α chains with different sequences (α1, α2, and α3) (i.e., type VI) [96].

Among these collagens, type I and type III collagens are important for wound healing [10,27], and both have fibrillar structures [97]. Type I collagens are the most profuse and ubiquitous of the collagens in the connective tissues [97]. Evidence has shown that the deposition of different types of collagens may cause diverse wound healing [7,8,9,10]. Type I collagen is dominant in normal wounds; by contrast, in hypertrophic scar or keloid scar, type III is dominant [4].

A myriad of factors stringently control the formation and degradation of collagens [98,99,100,101,102,103,104,105]. Among these, matrix metalloproteinases (MMPs), a family of zinc dependent proteases, are crucial [97]. These MMPs involve the tight control of ECM not only for an initial step of wound healing—inflammation—but also for the end step of wound healing, remodeling over time [99]. The interaction of the keratinocytes with collagen I, in animal models, triggers MMP-1 expression immediately when tissue insult occurs, and the basal keratinocytes at the epithelial front secretes MMP-1, which cleaves the provisional matrix, paving the path for the proper migration of these rapidly proliferating cells at the distal end [98]. This highest level of MMP-1 at day one gradually will decrease to basal level towards the completion of re-epithelialization. Persistent high levels of MMP-1 (collagenase 1) result in impairing wound healing [98]. This abnormal over-activity of MMP-1 is also supported by other studies [99].

One study showed that a magnitude of a 116-fold increase in the average protease activity is found in abnormal wound exudates when compared to normal acute wound exudates [97]. The expressions of the other MMPs, such as MMP-8 and MMP-13, are also disrupted in poor wound healing. MMP-8 (collagenase 2) has a stronger affinity toward type I collagen; therefore, the overexpression and activation of MMP-8 is directly involved in the pathogenesis of chronic non-healing wounds [98,99,100,101,102,103,104]. Absent expression of MMP-13 (collagenase 3) in the epidermis and overexpression of MMP-13 in non-healed wounds also suggests that the expression of several MMPs are derailed both at mRNA and protein levels in abnormal wound healing [98].

MMPs also regulate cell-cell and cell-matrix interactions through modulating and releasing cytokines, growth factors, and other biological active fragments that are sequestered in the ECM [100]. Growing evidence has convincingly identified select MMPs in membrane-type and intracellular compartments with unexpected physiological and pathological roles [98,99]. Membrane-type MMPs involve sheddase activities, collagenolysis, bacterial killing, and intracellular trafficking reaching as far as the nucleus. These membrane-type MMPs may also support pericellular proteolysis and endocytosis [100]. The above described research supports the important role of MMPs in wound healing, because MMPs are not only responsible for the direct degradation of ECM molecules, but are also key modulators of cardinal bioactive factors [102,103].

Coinciding with the dysregulation of MMPs, the tight regulation of endogenous inhibitors, tissue inhibitors of metalloproteinases (TIMPs), is also reported to be involved in wound healing [101,102]. These TIMPs directly regulate MMP activity and the MMPs/TIMPs balance can determine the net MMP activity, ECM turnover, and tissue remodeling, including wound healing.

The successful remodeling phase of wound healing depends on type I collagen in place of the original type III collagen. Type III collagens have more “flexible” potential cleavage sites than type I, and thus are more susceptible to hydrolysis by a variety of MMPs [97]. The classical collagenases include MMP-1, MMP-8, and MMP-13, based on efficiently catalyzing collagen hydrolysis ability [94]. Membrane-type I MMP prefers type I collagen, and the activity against type I collagen is 6.5 times that of type III collagens [97]. Since clear evidence shows that a single MMP cannot be unequivocally labeled as “good” or “bad”, because the net result of proteolytic activity should be dependent on situations when considering wound healing in general [104], it is not surprising that dysregulation in any protease function that affects ECM homeostasis might contribute to poor wound healing.

## 7. Conclusions

A full understanding of cutaneous estrogen synthesis and signaling is essential for future estrogen-based pharmacological manipulations of wound healing. Existing clinical estrogen-like compounds (e.g., tamoxifen, raloxifene, phytoestrogens, and genistein) are also known to influence the wound healing process [105], suggesting that the effects of estrogen on wound healing are complicated and worthy of further investigation. Translation of these estrogen and estrogen-like compound-mediated pathways from the bench to the clinic remains a promising proposition.

## Abbreviations

The following abbreviations are used in this manuscript:

GF	growth factor
ECM	extracellular matrix
TGF-β	transforming growth factor-β
DHEA	dehydroepiandrosterone
SERMs	selective estrogen receptor modulators
ER	estrogen receptor
Cyr61	cysteine-rich 61
IL	interleukin
GPER1	G-protein-coupled ER1
GRP30	G-protein-coupled receptor 30
Arg1	arginase 1
TNF	tumor necrosis factor
MIF	macrophage migration inhibitory factor
RR	relative risk
CI	confidence interval
MMPs	metalloproteinases
TIMPs	tissue inhibitors of metalloproteinases
siRNA	small interfering RNA
MAP	mitogen-activated kinase (MAP)
PI3K	phosphatidylinositol-3-kinase
miRNAs	microRNAs
CXCL-8	C-X-C motif chemokine ligand 8
IFN-γ	interferon-γ
